# Effects of positive end-expiratory pressure on cerebral hemodynamics in acute brain injury patients

**DOI:** 10.3389/fphys.2023.1139658

**Published:** 2023-05-02

**Authors:** Alberto Giardina, Danilo Cardim, Pietro Ciliberti, Denise Battaglini, Lorenzo Ball, Magdalena Kasprowicz, Erta Beqiri, Peter Smielewski, Marek Czosnyka, Shirin Frisvold, Matjaž Groznik, Paolo Pelosi, Chiara Robba

**Affiliations:** ^1^ Dipartimento di Scienze Chirurgiche e Diagnostiche, University of Genoa, Genova, Italy; ^2^ Department of Neurology, University of Texas Southwestern Medical Center, Dallas, TX, United States; ^3^ Institute for Exercise and Environmental Medicine, Texas Health Presbyterian Hospital, Dallas, TX, United States; ^4^ IRCCS Policlinico San Martino, Genova, Italy; ^5^ Department of Biomedical Engineering, Faculty of Fundamental Problems of Technology, Wroclaw University of Science and Technology, Wroclaw, Poland; ^6^ Brain Physics Laboratory, Department of Clinical Neurosciences, University of Cambridge, Cambridge, United Kingdom; ^7^ Institute of Electronic Systems, Warsaw University of Technology, Warsaw, Poland; ^8^ Anesthesia and Intensive Care, University Hospital of Northern Norway, Tromsø, Norway; ^9^ Traumatology Department of the University Clinical Center Ljubljana, Ljubljana, Slovenia

**Keywords:** positive end-expiratory pressure, intracranial pressure, cerebral autoregulation, acute brain injury, mechanical ventilation

## Abstract

**Background:** Cerebral autoregulation is the mechanism that allows to maintain the stability of cerebral blood flow despite changes in cerebral perfusion pressure. Maneuvers which increase intrathoracic pressure, such as the application of positive end-expiratory pressure (PEEP), have been always challenged in brain injured patients for the risk of increasing intracranial pressure (ICP) and altering autoregulation. The primary aim of this study is to assess the effect of PEEP increase (from 5 to 15 cmH_2_O) on cerebral autoregulation. Secondary aims include the effect of PEEP increase on ICP and cerebral oxygenation.

**Material and Methods:** Prospective, observational study including adult mechanically ventilated patients with acute brain injury requiring invasive ICP monitoring and undergoing multimodal neuromonitoring including ICP, cerebral perfusion pressure (CPP) and cerebral oxygenation parameters obtained with near-infrared spectroscopy (NIRS), and an index which expresses cerebral autoregulation (PRx). Additionally, values of arterial blood gases were analyzed at PEEP of 5 and 15 cmH_2_O. Results are expressed as median (interquartile range).

**Results:** Twenty-five patients were included in this study. The median age was 65 years (46–73). PEEP increase from 5 to 15 cmH_2_O did not lead to worsened autoregulation (PRx, from 0.17 (−0.003–0.28) to 0.18 (0.01-0.24), *p* = 0.83). Although ICP and CPP changed significantly (ICP: 11.11 (6.73–15.63) to 13.43 (6.8–16.87) mm Hg, *p* = 0.003, and CPP: 72.94 (59.19–84) to 66.22 (58.91–78.41) mm Hg, *p* = 0.004), these parameters did not reach clinically relevant levels. No significant changes in relevant cerebral oxygenation parameters were observed.

**Conclusion:** Slow and gradual increases of PEEP did not alter cerebral autoregulation, ICP, CPP and cerebral oxygenation to levels triggering clinical interventions in acute brain injury patients.

## Introduction

A large number of patients with brain damage require mechanical ventilation when admitted to the intensive care unit (ICU). The goal of mechanical ventilation is to optimize oxygen delivery and minimize lung and brain injury (Frisvold et al., 2019). The use of lung-protective ventilation strategies has been shown to reduce morbidity and mortality in critically ill patients (Sutherasan et al., 2014; Serpa Neto et al., 2015; Simonis et al., 2018). However, no specific recommendations are available on the optimal levels of positive end expiratory pressure (PEEP) to be applied in patients with acute brain injury (Robba et al., 2020).

PEEP can potentially cause an increase in intrathoracic pressures leading to a reduction in cerebral venous outflow and a consequent increase in intracranial pressure (ICP) and hemodynamic instability with reduction of cerebral perfusion pressure (CPP). On the other hand, alveolar overdistension can lead to an increase in arterial partial pressure of carbon dioxide (PaCO_2_) resulting in cerebral vasodilation. Thus, strategies involving the use of elevated PEEP are still controversial in brain-injured patients (Nemer et al., 2011; Borsellino et al., 2016; Robba et al., 2020). Previous studies from our group (Robba et al., 2021a; Robba et al., 2022) demonstrated that even high testing values of PEEP can be safe for ICP, as long as it promotes lung recruitment and improves lung compliance without causing hypotension. However, the effects of PEEP increase on more complex cerebral parameters such as cerebral autoregulation or cerebral oxygenation have not been completely elucidated. Assessment of these parameters is gaining particular interest in the context of multimodal monitoring of neurocritical care patients, as autoregulation impairment and brain hypoxia can reduce the tolerance to increased ICP, and can be associated with worse outcomes (Aries et al., 2012; Tas et al., 2021).

We therefore conducted a prospective observational study with the objective to evaluate cerebral hemodynamics including cerebral autoregulation, cerebral oxygenation and ICP using a brain multimodal monitoring approach at two different levels of PEEP in a cohort of acute brain injured patients.

## Methods

This is a prospective, observational study including adult mechanically ventilated brain injured patients requiring invasive ICP monitoring. The local ethics review board (Comitato Etico Regione Liguria, protocol n. CER Liguria: 23/2020) approved this study, and written consent was obtained from patients’ next of kin. We adhered to the “Strengthening the Reporting of Observational Studies in Epidemiology (STROBE)” statement guidelines for observational cohort studies (von Elm et al., 2014) (Additional file 1: ESM Supplementary Table S1).

### Inclusion and exclusion criteria

Patients were screened for inclusion from 1 February 2021 to 1 September 2022 at San Martino Policlinic Hospital, IRCCS for Oncology and Neurosciences, Genoa, Italy, and were considered eligible if they were 1) older than 18 years-old; 2) admitted to ICU for acute brain injury, i.e., traumatic brain injury (TBI), subarachnoid hemorrhage (SAH) or intracranial hemorrhage (ICH); 3) required invasive ICP monitoring and mechanical ventilation at ICU admission, and 4) underwent multimodal neuromonitoring (according to clinical severity and based on bed availability) including ICP, cerebral autoregulation and cerebral oxygenation during a PEEP test from 5 to 15 cmH_2_O. Our unit is a mixed general and neuro-ICU, composed of 28 level 3 beds, of which 12 are equipped with ICM + software. Patients without multimodal neuromonitoring, not mechanically ventilated or those patients whose next of kins refused to agree about inclusion in the study were excluded.

### Data collection

Electronic medical records were used to collect patients’ information, and included admission demographics (e.g., age, sex, body mass index (BMI), pre-injury comorbidities (respiratory, cardiovascular, liver and kidney disease, cancer, diabetes mellitus and hypertension), reason for ICU admission (TBI, SAH, ICH), neurological status at admission as for Glasgow coma scale (GCS) and pupils characteristics (reactivity, iso or anisocoria), ICU complications (acute distress respiratory syndrome (ARDS), ventilator-associated pneumoniae, acute kidney injury, sepsis, vasospasm) and patients’ clinical outcomes, such as ICU length of stay, number of days with mechanical ventilation, ICU mortality and neurological status (as for Glasgow Outcome Score-Extended (GOSE)), type of ICP monitoring (intraparenchymal or external ventricular drain).

Data on ventilatory mechanics, i.e., tidal volume, plateau pressure, and arterial blood gases (partial pressure of oxygen (PaO_2_) and of carbon dioxide (PaCO_2_) and multimodal neuromonitoring data before and after PEEP test were also collected.

### Patient’s clinical management

In the ICU, patients were sedated with propofol (3–6 mg/kg/h) and/or midazolam (0.03–0.2 mg/kg/h) and fentanyl (0.1–0.8 μg/kg/min), intubated and mechanically ventilated in pressure or volume-controlled ventilation. Tidal volume was targeted to 6–8 mL per kg of predicted body weight (PBW), but higher values of tidal volume were tolerated, if driving pressure was maintained below 15 cmH_2_O. Invasive ICP monitoring was inserted according to our local policies and clinical practice, following latest Brain Trauma Foundation Guidelines (Carney et al., 2017), as well as patients’ clinical management.

The decision to perform a PEEP test was based on clinicians’ evaluation if optimization of mechanical ventilation was required, to set the best PEEP level, according to local protocol (Robba et al., 2022) and our previous experience.

PEEP test was performed in volume-controlled ventilation in all patients, without using neuromuscular blockade. PEEP was slowly increased from 5 to 15 cmH_2_O, about 2 cmH_2_O every minute, evaluating the changes in respiratory mechanics and cerebral hemodynamics, with the aim to set the best PEEP value. Our local protocol is based on our previous clinical experience which suggested that these PEEP values are safe in brain injured patients (Robba et al., 2021a), and on a recent consensus on mechanical ventilation in brain injured patients, which suggested to use the same level of PEEP applied in the non-brain injured population (Robba et al., 2020).

Data were obtained at PEEP of 5 (T0) and at 15 cmH_2_O (T1) after allowing 5 min for stabilization, as previously described (Cressoni et al., 2017), and the chosen levels of PEEP (5 and 15 cmH_2_O) represent the standard levels of PEEP used to estimate response to PEEP in general ICU patients (Gattinoni et al., 2006).

Data collected at T0 and T1 included respiratory mechanics and arterial blood gases parameters including arterial partial pressure of oxygen (PaO_2_) and of carbon dioxide (PaCO_2_), tidal volume (VT), plateau pressure (Pplat), respiratory system compliance (CRS), driving pressure (DP), arterial saturation of oxygen (SaO_2_), as well as neuromonitoring data, i.e., ICP, pressure reactivity index (PRx, as means for cerebral autoregulation assessment), and data on cerebral oxygenation derived from near-infrared spectroscopy (NIRS).

### Multimodal neuromonitoring

Intracranial pressure was monitored continuously with a transducer into the brain parenchymal space or through an external cerebral fluid shunt, according to clinical practice. Arterial blood pressure was monitored in the radial or femoral artery zeroed at the level of the right atrium (Baxter Healthcare, CA, United States; Sidcup, United Kingdom). In patients with head elevation, no corrections were made for hydrostatic pressure differences.

For the assessment of cerebral oxygenation, we used non-invasive continuous regional cerebral oxygen saturation using the Root^®^ with O_3_^®^ regional oximetry device (Masimo, Irvine, CA, United States), with a bilateral sensor applied in the frontotemporal region. Final values of cerebral oxygenation measurements at T0 and T1 were calculated as the mean between single instant measurements obtained from the right and left frontotemporal sensors. Different parameters of cerebral oxygenation can be obtained from this monitor: a) rSO_2_, which represents the regional cerebral oxygen saturation, and is derived as the ratio of the concentration of oxyhemoglobin (O_2_Hb) and total hemoglobin (cHb = O_2_Hb + HHb, where HHb is deoxyhemoglobin); b) ΔO_2_Hbi, which is an index associated with changes of concentration of oxyhemoglobin, thus representing predominantly changes in the arterial component of regional oxygen saturation; c) ΔHHbi, an index reflecting changes in concentration of deoxyhemoglobin, approximately representing changes in the venous component of the oxygen saturation; d) ΔcHbi, an index representing the sum of ΔO_2_Hbi and ΔHHbi components (total hemoglobin content) (Gattinoni et al., 2006; Robba et al., 2021a).

All continuous physiological data were collected simultaneously and analyzed using ICM+ (Cambridge Enterprise, Cambridge, United Kingdom, https://icmplus.neurosurg.cam.ac.uk) (Smielewski et al., 2005), a clinical research software which can provide real-time analysis of multimodal monitoring modalities at the patient’s bedside. Data collected with ICM+ were sampled at 100 Hz. Artifacts were visually inspected and manually removed from the data time series using artifact removal tools on ICM+. Typical artifacts in the data consisted of spikes in ICP due to suction, or arterial line flushes. Cerebral autoregulation assessed through PRx and calculated over a 5-min moving window as the Pearson correlation of 30 consecutive 10-s average values of ABP and ICP as previously described (Czosnyka et al., 1997). A preserved autoregulation was defined as values of PRx below 0.05 averaged over a 10-min period, whereas higher values (above 0.25) are defined as altered autoregulation (Sorrentino et al., 2012). PRx was calculated from T0 (averaged value of a 10-min period before PEEP increase) and T1 (averaged value of a 10-min period immediately after PEEP increase to 15 cmH_2_O) periods.

### Statistical analysis

The Shapiro-Wilk test was used to test the normality of the distribution of the variables. Continuous variables are reported as median and interquartile range (IQR = 25th −75th percentiles). Comparisons between different variables at T0 and T1 were made by repeated measures (paired) *t*-test for normally distributed variables, while non-normally distributed variables were compared by paired Wilcoxon signed-rank test. Graphical representations of these comparisons are presented as boxplots. Dependent variables were expressed as a change from baseline (T0) in absolute terms (Δ change = T1-T0). The correlations coefficients between systemic and the different neuromonitoring variables were verified using Pearson’s or Spearman’s method, for parametric and non-parametric variables, respectively. All statistical analyses were performed using RStudio software (version 4.1.1). A *p*-value <0.05 was considered statistically significant.

## Results

During the study period, a total of 110 patients were considered for inclusion. Fifty-two patients were excluded as they did not undergo multimodal neuromonitoring and 33 patients were not allocated to a specific bed with ICM+. A final number of 25 patients were included in the analysis. The characteristics of the patients are presented in Table 1. 60% were male, and the median age was 64.7 years (45.9-73.2). Thirteen patients (52%) were admitted for TBI, 7 (28%) for SAH, and 5 (20%) patients for ICH. Six patients (24%) had a history of hypertension. At ICU discharge, median GOSE was 3 (1.8-4.0), and 5 patients died (20%).

**TABLE 1:** Patients demographics, characteristics, intensive care unit (ICU) complications and patient outcomes.

General characteristics	
Age, years, median [IQR]	64.7 [45.9; 73.2]
BMI, kg/m^2^, median [IQR]	25.0 [23.4; 26.1]
Gender, male/female, n (%)	15/10 (60/40)
Comorbidities	
None, n (%)	6 (24)
≥2 comorbidities, n (%)	9 (36)
Smoke habits, n (%)	5 (20)
Hypertension, n (%)	6 (24)
Diabetes, n (%)	1 (4)
Other endocrine/metabolic disease, n (%)	3 (12)
Alcohol and/or drugs abuse, n (%)	1 (4)
Kidney injury, n (%)	1 (4)
Liver injury, n (%)	1 (4)
Depression and/or anxiety, n (%)	1 (4)
Cardiovascular disease, n (%)	1 (4)
Neurological disease and severity	
Out-of-hospital GCS, points, median [IQR]	6 [3; 11.5]
Anisocoria, n (%)	8 (32)
Type of brain injury	
SAH, n (%)	7 (28)
TBI, n (%)	13 (52)
ICH, n (%)	5 (20)
Type of invasive monitoring	
EVD, n (%)	9 (36)
Intraparenchymal bold, n (%)	16 (64)
TBI, Marshall classification	
I	0 (0)
II	0 (0)
III	5 (38.4)
IV	4 (30.8)
V	4 (30.8)
VI	0 (0)
SAH, Fisher classification	
I	0 (0)
II	0 (0)
III	0 (0)
IV	7 (100)
ICU outcomes and complications	
GOSE, points, median [IQR]	3.0 [1.8; 4.0]
ICU complications	
≥2 complications, n (%)	8 (32)
None, n (%)	7 (28)
Septicemia, n (%)	13 (52)
VAP, n (%)	8 (32)
Acute kidney injury, n (%)	1 (4)
Meningitis, n (%)	1 (4)
Epileptic crisis, n (%)	2 (8)
Hydrocephalus, n (%)	1 (4)
Vasospasm after SAH, n (%)	1 (4)
ICU-LOS, days, median [IQR]	32.0 [20.0; 52.5]
MV duration, days, median [IQR}	16.0 [9.5; 26.5]
Mortality, n (%)	5.0 (20)

IQR, interquartile range; n, number; BMI, body mass index; PBW, predicted body weight; ICU, intensive care unit; TBI, traumatic brain injury; SAH, subarachnoid hemorrhage; ICH, intracranial hemorrhage; GCS, glasgow coma scale; ICP, intracranial pressure; EVD, external ventricular drain; GOSE, glasgow outcome score extended; ICU-LOS, intensive care unit length of stay; MV, mechanical ventilation.

### Effect of PEEP test on cerebral and systemic factors

**FIGURE 1:**
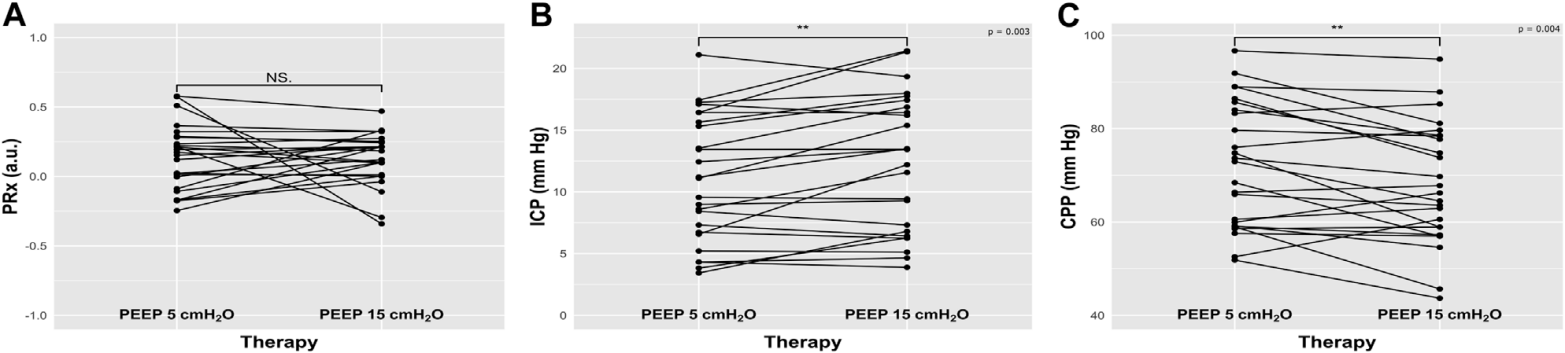
Plots representing the effect of increased positive end-expiratory pressure (PEEP) on cerebral autoregulation measured with pressure reactivity test (PRx), intracranial pressure (ICP), and cerebral perfusion pressure (CPP) from baseline. NS: not statistically significant; **: *p* < 0.01.

After PEEP test, changes in PRx were not statistically significant, from 0.17 (−0.003-0.28) to 0.18 (0.01–0.24), *p* = 0.83, Table 2; Figure 1).

**TABLE 2:** Neuromonitoring and systemic parameters at baseline (PEEP 5 cmH_2_O) and post-PEEP test (PEEP 15 cmH_2_O) (median (interquartile range)).

	PEEP 5 cmH_2_O	PEEP 15 cmH_2_O	Δ change	*p*-value
ICP	11.11 (6.73–15.65)	13.43 (6.80–16.87)	0.72 (−0.13–2.97)	**0.003**
CPP	72.94 (59.19–84.00)	66.22 (58.91–78.41)	−2.36 (−10.73–0.35)	**0.004**
ABP	83.38 (74.69–92.24)	78 (72–87)	−1.90 (−6.14–0.34)	**0.02**
PRx	0.17 (−0.003–0.28)	0.18 (0.01–0.24)	0.002 (−0.05–0.14)	0.83
rSO_2_	59 (55.78–62.81)	59 (55.70–62.23)	−0.02 (−0.59–0.16)	0.43
ΔO_2_Hbi	3.80 (2.10–4.60)	3.60 (2.10–6.21)	0.90 (−0.60–2.70)	0.08
ΔHHbi	1.50 (−0.19–3.30)	2.80 (1.73–4.53)	0.40 (−0.08–1.79)	0.055
ΔcHbi	4.20 (2.92–6.90)	5.98 (3.75–11.01)	1.50 (0.0–3.06)	**0.02**
SpO_2_	97 (96.00–97.42)	98 (96.00–99.29)	1 (-1–2)	0.17
PaO_2_	87 (79–93)	93 (88–98)	5 (1–13)	**0.01**
PaCO_2_	40 (39–43)	41 (38–43)	0 (-1–1)	0.93
VT	500 (479.50–535.00)	460 (431.00–493.50)	−40 (−70-(-30))	**0.003**
Pplat	20 (18–22)	26 (24–28)	6 (4–8)	**<0.0001**
RR	20 (18–22)	21 (18–22)	0 (0–1)	0.32
CRS	31.88 (30.24–37.88)	41.29 (35.51–49.69)	8.12 (2.33–14.74)	**<0.001**
DP	15 (13–17)	11 (9–13)	−4 (-6-(-2))	**<0.0001**

Abbreviations: ICP (mm Hg), intracranial pressure; CPP (mm Hg), cerebral perfusion pressure; ABP (mm Hg), arterial blood pressure; PRx (a.u.), pressure reactivity index; rSO_2_, regional cerebral oxygen saturation (%); ΔHHbi (μM.cm), changes in concentration of deoxygenated hemoglobin (of the total rSO_2_); ΔO_2_Hbi (μM.cm), changes in concentration of oxygenated hemoglobin (of the total rSO_2_); ΔcHbi (μM.cm), changes in concentration of total hemoglobin; SpO_2_ (%), saturation percent of oxygen; PaO_2_ (mm Hg), arterial partial pressure of oxygen; PaCO_2_ (mm Hg), arterial partial pressure of carbon dioxide; VT (mL), tidal volume; Pplat (cmH_2_O), plateau pressure; RR, respiratory rate; CRS (mL/cmH_2_O), compliance of respiratory system; DP (cmH_2_O), driving pressure. Statistically significant values are presented in bold.

**FIGURE 2:**
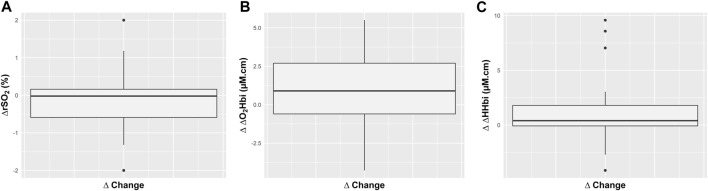
Boxplots representing the effect of increased positive end-expiratory pressure (PEEP) on absolute changes (∆) in regional cerebral oxygen saturation (rSO_2_), and in the arterial (ΔO_2_Hbi) and venous (ΔHHbi) components of cerebral oxygenation.

ICP significantly changed (from 11.11 (6.73–15.63) mm Hg to 13.43 (6.8–16.87) mm Hg, *p* = 0.003, as well as ABP and CPP (from 83.38 (74.69–92.24) to 78 (72–87) mm Hg, *p* = 0.02, and from 72.94 (59.19–84) mm Hg to 66.22 (58.91–78.41) mm Hg, *p* = 0.004, respectively) (Table 2; Figure 1). On the other hand, no changes in cerebral oxygenation parameters were observed from T0 to T1 (Table 2; Figure 2), apart from the changes in total hemoglobin content (ΔcHbi) (Table 2).

Systemic PaO_2_ also increased, from 87 (79–93) to 93 (88-98) mm Hg, *p* = 0.03), without causing changes in PaCO_2_ (from 40 (39-43) to 41 (38–43) mm Hg, *p* = 0.89) (Table 2).

Considering ventilatory settings, PEEP increase led to reduced tidal volume (500 (479–535) to 460 (431.00–493.50) mL, *p* = 0.003), increased plateau pressure (20 (18-22) to 26 (24-28) cmH_2_O, *p* < 0.0001), reduced driving pressure (15 (13–17) to 11 (9–13) cmH_2_O, *p* < 0.0001), and improvement of respiratory system compliance (from 31.88 (30.24–37.88) to 41.29 (35.51–49.69) mL/cmH_2_O, *p* < 0.001) (Table 2).

**FIGURE 3:**
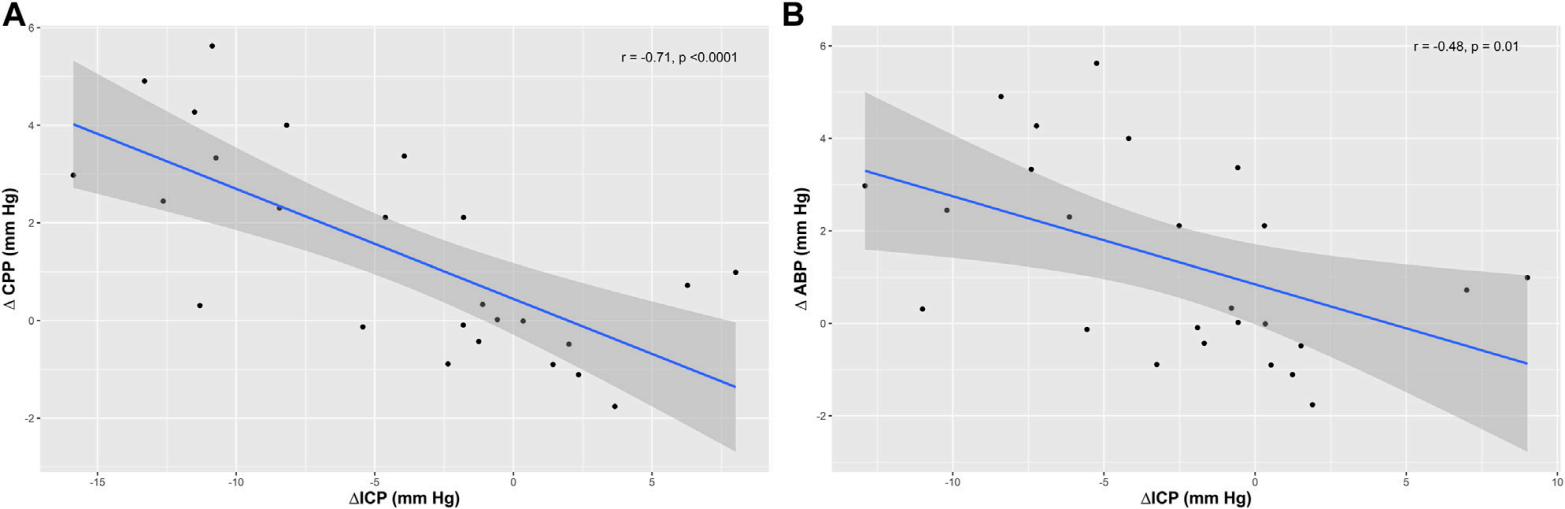
Scatter plots showing the correlation (r) between changes in intracranial pressure (ΔICP) and **(A)** cerebral perfusion pressure (ΔCPP), and **(B)** mean arterial blood pressure.

No significant correlations were observed between the changes in ICP and other parameters (Table 3), except with CPP and ABP (r = −0.71, *p* < 0.0001, and r = −0.48, *p* = 0.01) (Figure 3). Changes in compliance of respiratory system were correlated with ΔO_2_Hbi (r = 0.44, *p* = 0.03).

**TABLE 3:** Correlation matrix between changes (Δ) in systemic, ventilatory and neuromonitoring parameters.

	ΔICP	ΔCPP	ΔABP	ΔPRx	ΔSpO_2_	ΔrSO_2_	ΔO_2_Hbi	ΔHHbi	ΔcHbi	ΔPaO_2_	ΔPaCO_2_	ΔPplat	ΔVT	ΔRR	ΔCRS	ΔDP
ΔICP																
r	-	−0.71	−0.48	0.08*	0.28	−0.07	0.07	−0.01*	0.04*	−0.05	−0.06	−0.19*	0.05*	−0.14*	0.13*	−0.19*
*p*-value	-	**<0.0001**	**0.01**	0.72	0.18	0.73	0.74	0.96	0.87	0.80	0.76	0.36	0.82	0.52	0.54	0.36
**ΔCPP**																
r	−0.71	-	0.96	−0.17	0.41	0.25	0.07	−0.16*	−0.07*	−0.09	0.17	0.22*	−0.10*	−0.23*	−0.15*	0.22*
*p*-value	**<0.0001**	-	**<0.0001**	0.42	**0.04**	0.22	0.74	0.43	0.78	0.68	0.42	0.29	0.64	0.27	0.46	0.29
**ΔABP**																
r	−0.48	0.96	-	−0.15	0.39	0.29	0.11	−0.26*	−0.06*	−0.13	0.18	0.19*	−0.05*	−0.28*	−0.07*	0.19*
*p*-value	**0.01**	**<0.0001**	-	0.48	**0.05**	0.16	0.59	0.21	0.80	0.54	0.38	0.36	0.80	0.18	0.74	0.36
**ΔPRx**																
r	0.08*	−0.17*	−0.15*	-	0.32*	−0.36*	0.06*	0.04*	0.13*	0.14*	−0.37*	0.01*	0.18*	0.08*	0.04*	0.01*
*p*-value	0.72	0.42	0.48	-	0.12	0.07	0.77	0.87	0.52	0.52	0.07	0.94	0.39	0.69	0.87	0.94
**ΔSpO**_**2**_																
r	0.28	0.41	0.39	0.32*	-	−0.18	0.22	−0.23*	0.30*	0.27	0.31	0.36*	−0.13*	−0.09*	−0.20*	0.36*
*p*-value	0.18	**0.04**	0.05	0.12	-	0.39	0.30	0.28	0.14	0.18	0.13	0.07	0.53	0.69	0.33	0.07
**ΔrSO**_**2**_																
r	−0.07	0.25	0.29	−0.36*	−0.18	-	0.28	0.22*	0.67*	0.07	0.32	−0.27*	−0.17*	−0.10*	0.31*	−0.27*
*p*-value	0.73	0.22	0.16	0.07	0.39	-	0.17	0.29	**<0.001**	0.74	0.11	0.19	0.40	0.62	0.13	0.19
**ΔO**_**2**_ **Hbi**																
r	0.07	0.07	0.11	0.06*	0.22	0.28	-	−0.07*	0.59*	0.05	−0.26	−0.17*	−0.21*	−0.26*	0.44*	−0.17*
*p*-value	0.74	0.74	0.59	0.77	0.30	0.17	-	0.74	**0.002**	0.81	0.21	0.43	0.30	0.21	**0.03**	0.43
**ΔHHbi**																
r	−0.01*	−0.16*	−0.26*	0.04*	−0.23*	0.22*	−0.07*	-	0.09*	0.02*	0.01*	0.13*	0.20*	−0.08*	−0.14*	0.13*
*p*-value	0.96	0.43	0.21	0.87	0.28	0.29	0.74	-	0.64	0.92	0.95	0.54	0.34	0.69	0.51	0.54
**ΔcHbi**																
r	0.04*	−0.07*	−0.06*	0.13*	0.13*	0.30*	0.67*	0.59*	-	0.09*	−0.26*	−0.05*	−0.08*	−0.15*	0.38*	−0.05*
*p*-value	0.87	0.78	0.80	0.52	0.52	0.14	**<0.001**	**0.002**	-	0.65	0.21	0.81	0.71	0.46	0.06	0.81
**ΔPaO**_**2**_																
r	−0.05	−0.09	−0.13	0.14*	0.27	0.07	0.05	0.02*	−0.09*	-	−0.06	−0.09*	0.12*	−0.06*	0.02*	−0.09*
*p*-value	0.80	0.68	0.54	0.52	0.18	0.74	0.81	0.92	0.65	-	0.78	0.67	0.56	0.77	0.91	0.67
**ΔPaCO**_**2**_																
r	−0.06	0.17	0.18	−0.37*	0.31	0.32	−0.26	0.01*	−0.26*	−0.06	-	0.04*	0.19*	−0.19*	−0.22*	0.04*
*p*-value	0.76	0.42	0.38	0.07	0.13	0.11	0.21	0.95	0.21	0.78	-	0.85	0.35	0.37	0.29	0.85

r: correlation coefficient. * represents Spearman correlation coefficients; the remaining values represent Pearson correlation coefficients.

ICP: intracranial pressure; CPP: cerebral perfusion pressure; PRx: pressure reactivity index; rSO_2_: regional tissue oxygen saturation; ∆O_2_Hbi: index representing the change in the oxyhemoglobin of the regional tissue oxygen saturation; ∆HHbi: index representing the change in the deoxyhemoglobin of the regional tissue oxygen saturation; ∆HHbi: index representing the change in total hemoglobin; SpO_2_: systemic oxygen saturation; PaO_2_: partial pressure of O_2_. Statistically significant values are presented in bold.

## Discussion

In our cohort of mechanically ventilated brain injured patients we found that: 1) ICP, CPP and ABP significantly changed after the PEEP test, however, PEEP increase did not worsen PRx; 2) No changes in cerebral oxygenation parameters related to arterial or venous components were observed; 3) There was an increase of systemic PaO_2_, without causing changes in PaCO_2_. Respiratory system compliance was improved.

The use of high PEEP has been challenged in the ABI population, as it can potentially increase intrathoracic pressure, reduce jugular vein outflow, decrease venous return, and lead to a drop in cardiac output and blood pressure. This may potentially cause hemodynamic instability with consequent detrimental effects on CPP and cerebral blood flow. When cerebral autoregulation is intact, extreme reduction of CPP can lead to cerebral vasodilation and an increase in cerebral blood volume that can potentially exacerbate ICP; on the other hand, when autoregulation is impaired, decreased CPP may lead to cerebral ischemia (Calviello et al., 2017). The transmission of PEEP into the thoracic cavity is variable and dependent on the properties of the chest wall and lungs. Some studies suggest that when the chest wall compliance is low, PEEP significantly increases intrathoracic pressure, and if PEEP does not lead to recruitment but causes alveolar hyperdistention, this can magnify the effect of PEEP and intrathoracic pressure on ICP (Burchiel et al., 1981; Robba et al., 2021a).

A multimodal approach for the assessment of cerebral hemodynamics, especially cerebral autoregulation, is therefore of great interest, and warrants clinical relevance in this context. We assessed a heterogeneous group of patients with ABI at fixed PEEP levels of 5 cmH_2_O and 15 cmH_2_O using hemodynamic parameters and recorded cerebral and respiratory function parameters. After the PEEP test, we observed an increase of PaO_2_ and improvement of CRS. PEEP increase in our cohort did not lead to increase of PaCO_2_ and the effect on cerebral physiological parameters was minimal, with no significative changes in autoregulation. These preliminary results suggest that an increase in PEEP within the proposed range might be a safe maneuver in patients with ABI, provided this improves respiratory mechanics and does not affect importantly hemodynamic status.

Survival after ABI is dependent on the control of intracranial hypertension and the provision of hemodynamic support to achieve an appropriate cerebral perfusion pressure (Robba et al., 2021b). The current Brain Trauma foundation Guidelines suggest targeting CPP between 60 and 70 mm Hg after TBI (Carney et al., 2017).

However, the idea of a single value or even a single range of CPP being suitable for the diverse group of ABI patients is an oversimplification. Age, comorbidities, and pre-injury arterial blood pressure are examples of factors likely to influence individual CPP targets, with elderly, hypertensive patients requiring a higher CPP compared with young, normotensive patients. In addition, in healthy, the ability to autoregulate occurs in a very wide CPP range, but after brain injury, this ability is impaired, and the risk of secondary damage increases dramatically (Menon, 1999).

The examination of continuous autoregulation and the definition of optimal CPP, which is the CPP at which each patient autoregulates at best, requires sophisticated signal analysis. ICM + bedside software and pressure reactivity index (PRx) have already been extensively validated in the literature and allow an individualized assessment of the effect of respiratory manipulations on the brain (Donnelly et al., 2015; Donnelly et al., 2018). PRx–which represents the between changes in arterial blood pressure and intracranial pressure offers a surrogate method for the continuous bedside estimation of global cerebral autoregulation, which has been suggested to be feasible and safe (Tas et al., 2021), and is considered at present the most accurate method for individualized autoregulation assessment (Needham et al., 2017; Depreitere et al., 2021).

A multimodal approach of respiratory and cerebral parameters can therefore help with a better definition of the best individualized PEEP value to be applied in order to promote improvement of respiratory mechanics without altering cerebral dynamics.

This study has several limitations that need to be mentioned. Firstly, this is a single center study with a small sample, and heterogeneity of included patients, which limit the generalization of our results. The posteriori use of the data as presented in this study setting (e.g., the need to wait for the recording and calculation of different parameters) can potentially delay the modification of interventions in clinical practice. Only two arbitrary levels of PEEP were investigated for technical reasons and patient safety concerns, and as for our local protocols. The application of this protocol may cause physiological changes in CPP after an increase in PEEP due to a decrease in ABP or increase in right pressures above ICP. High levels of PEEP are useful in ARDS patients in whom pulmonary compliance is low; in such patients the PEEP effects on cerebral hemodynamics likely differ from the changes observed in most patients. Known limitations of NIRS, such as the potential influence of extracranial contamination (particularly in HHb and O_2_Hb signals), the nature of HHb and HbO_2_ dependent on the unknown scattering coefficient likely differing on an individual basis, and the unknown contribution of venous and arterial components to the measured signals, particularly O_2_Hb, are potential confounders in our study. Furthermore, we did not assess any direct measures of CBF in these patients. Finally, all patients had relatively low ICP at the moment of measurement; moreover, we cannot exclude that different ventilator settings or the addition of a recruitment maneuver may have led to different results.

## Conclusion

In this study, PEEP increase led to improvement of respiratory system compliance, without affecting importantly systemic hemodynamics and PaCO_2_ values. This led to the absence of detrimental effects on cerebral autoregulation, cerebral oxygenation and intracranial pressure.

These results suggest that the use of augmented PEEP can be safe in acute brain injured patients. Our findings are in line with the current recommendations for this patient population, which suggests applying the same level of PEEP as in the general ICU population. This study also highlights the applicability and feasibility of a multimodal approach for the individualization of lung and cerebral management in future studies.

## Data Availability

The original contributions presented in the study are included in the article/Supplementary Material, further inquiries can be directed to the corresponding author.
